# Experiences, opportunities and challenges of implementing task shifting in underserved remote settings: the case of Kongwa district, central Tanzania

**DOI:** 10.1186/1472-698X-12-27

**Published:** 2012-11-02

**Authors:** Michael A Munga, Stella P Kilima, Prince Pius Mutalemwa, William J Kisoka, Mwelecele N Malecela

**Affiliations:** 1 Deparment of Monitoring and Evaluation, National Institute for Medical Research, P.O Box 9653, Dar es Salaam, Tanzania; 2 Department of Health Systems and Policy Research, National Institute for Medical Research, P.O Box 9653, Dar es Salaam, Tanzania; 3 National Institute for Medical Research, P.O Box 9653, Dar es Salaam, Tanzania

## Abstract

**Background:**

Tanzania is experiencing acute shortages of Health Workers (HWs), a situation which has forced health managers, especially in the underserved districts, to hastily cope with health workers’ shortages by adopting task shifting. This has however been due to limited options for dealing with the crisis of health personnel. There are on-going discussions in the country on whether to scale up task shifting as one of the strategies for addressing health personnel crisis. However, these discussions are not backed up by rigorous scientific evidence. The aim of this paper is two-fold. Firstly, to describe the current situation of implementing task shifting in the context of acute shortages of health workers and, secondly, to provide a descriptive account of the potential opportunities or benefits and the likely challenges which might ensue as a result of implementing task shifting.

**Methods:**

We employed in-depth interviews with informants at the district level and supplemented the information with additional interviews with informants at the national level. Interviews focussed on the informants’ practical experiences of implementing task shifting in their respective health facilities (district level) and their opinions regarding opportunities and challenges which might be associated with implementation of task shifting practices. At the national level, the main focus was on policy issues related to management of health personnel in the context of implementation of task shifting, in addition to seeking their opinions and perceptions regarding opportunities and challenges of implementing task shifting if formally adopted.

**Results:**

Task shifting has been in practice for many years in Tanzania and has been perceived as an inevitable coping mechanism due to limited options for addressing health personnel shortages in the country. Majority of informants had the concern that quality of services is likely to be affected if appropriate policy infrastructures are not in place before formalising tasks shifting. There was also a perception that implementation of task shifting has ensured access to services especially in underserved remote areas. Professional discontent and challenges related to the management of health personnel policies were also perceived as important issues to consider when implementing task shifting practices. Additional resources for additional training and supervisory tasks were also considered important in the implementation of task shifting in order to make it deliver much the same way as it is for conventional modalities of delivering care.

**Conclusions:**

Task shifting implementation occurs as an *ad hoc* coping mechanism to the existing shortages of health workers in many undeserved areas of the country, not just in the study site whose findings are reported in this paper. It is recommended that the most important thing to do now is not to determine whether task shifting is possible or effective but to define the limits of task shifting so as to reach a consensus on where it can have the strongest and most sustainable impact in the delivery of quality health services. Any action towards this end needs to be evidence-based.

## Background

Like many countries in the world, Tanzania is experiencing acute shortages of health workers, a situation which has forced health managers, especially in the underserved districts, to hastily cope with such problem by adopting task shifting. However, this has been due to limited options to deal with health personnel shortages. Moreover, there have been on-going discussions at various decision making levels in Tanzania regarding the most favourable and convenient ways of implementing the strategy, albeit as a short term solution. However, these discussions are not backed up by rigorous scientific evidence. It is thus crucial to generate such evidence in order to inform policy and practice. The aim of this paper is two-fold. Firstly, to describe the current status of implementing task shifting in the context of acute shortage of health workers. Secondly; the paper attempts to provide a descriptive account of the potential opportunities, benefits and the likely challenges which might ensue as a result of implementing task shifting.

In the literature, task shifting has been defined in different ways depending on various circumstances of health care settings. Almost unanimously, the literature consider task shifting as the process of delegation of tasks from skilled to low or unskilled health workers [1,2].The World Health Organization (WHO) defines task shifting as “the ‘rational’ re-distribution of tasks among health workforce teams” [3,4]. “Specific tasks are moved, where appropriate, from highly qualified health workers to health workers with shorter training and fewer qualifications in order to make more efficient use of available human resources for health” [3,4]. Delegation of tasks to community lay workers has also been prevalent in many sub-Saharan African countries [4,5].

In almost all of its typologies, task shifting is not a new concept. In the last few years, this concept and its practice had re-emerged to be at the centre stage of global debates and had now been considered to be one of the potential strategies to address acute shortages and unmet needs for health workers in the health care systems of many countries. Task shifting has seen a welcome re-emphasis by the World Health Organization (WHO) whereby in 2010 it had issued global recommendations (see Task shifting pre-conditions section below) on how to design, manage, monitor and evaluate task shifting practices [3]. The main perceived aim of task shifting is to improve access to health care services in places with acute shortages of health personnel without compromising its quality [3].

Task-shifting has been shown to be effective in high-income countries whereby appropriately trained and well supervised lower-level cadres perform delegated tasks well or better than the trained ones [1,6,7]. Promising successes had been documented especially in relation to delivery of the HIV/AIDS interventions [5,8,9]. Despite evidence of task shifting’s cost effectiveness from other countries, there is little documented information in Tanzania regarding implementation of task shifting, notwithstanding the fact that it has been in practice for many years in the country. In addition, there is virtually no evidence on the potential benefits, opportunities and challenges which may be associated with implementing task shifting in the country. The World Health Organization has recently engaged a ‘speed’ gear towards advising countries to consider scaling up task shifting practices by offering a range of recommendations regarding optimal ways for its effective implementation. In many instances, emphasis has been placed on quality assurance issues as they are related to health services delivery in the context of task shifting [3,5].

There had been series of discussions by researchers and policy makers in Tanzania regarding task shifting as an innovative strategy to address health workforce shortages. However, the depth and breadth of these discussions are apparently narrow due to lack of rigorous scientific evidence. In July 2008 for example, the Tanzanian Ministry of Health and Social Welfare (MOHSW) in collaboration with the World Health Organization (WHO) organised a three-day national stakeholders’ consultative meeting to discuss issues related to task shifting, particularly its relevance in the context of Tanzania’s HRH situation. [9].The main objectives of that meeting were:

• To share WHO guidelines and global recommendations on task shifting

• To explain to HRH stakeholders the rationale for adopting task shifting and share relevant country experiences

• To mobilize stakeholders’ efforts and resources in order to assess how widespread of current task shifting is practiced in Tanzania

• To outline key steps for developing and implementing a national task shifting action plan (using WHO generic guidelines adapted to suite the Tanzanian context.

Among the resolutions reached in this meeting were a) to undertake a stakeholders’ analysis by focusing on the implementation of task shifting in Tanzania; b) to identify priority programmes^a^, which currently implement task shifting in response to shortage of health personnel required to implement their programme activities and c) to undertake a desk review in order to document and analyse task shifting experiences as implemented by different disease programmes. Up to the time of writing this paper, there was no documented evidence on how far the above recommendations were implemented. The findings reported in this paper provide one of those few resources given a weak knowledge base on the subject. This information is quite crucial for informing policy makers and programme planners about the current situation of task shifting implementation in the country’s public health care system. The findings will also add to the existing (albeit, weak) evidence base which is needed to back-up the on-going discussions about whether formalised task shifting in Tanzania is needed and whether it is feasible to ultimately deliver what is meant to deliver without compromising access and quality of health care services.

Against this background, the rest of the paper is organised as follows. The following section presents the context by describing the Tanzanian socioeconomic, health and demographic indicators, organization of the country’s health care system and health workforce situation and; reflects this with the need to implement task-shifting. This is followed by a description of the methodological approach. Subsequent sections present findings, discussion of the main findings and methodological limitations. Conclusions and recommendations are presented at the end of the paper.

### The context

Tanzania is one of the poorest countries in the world characterised by enormous shortage of health workers. The World Health Organization reports that the country has 0.02 physicians per 1000 population, the lowest in the world [10]. A recent analysis of the HRH situation indicated that there are 46,896 total health workers in Tanzania and are distributed as shown in Table 1 below [11]. On average, there are 1.4 total health workers per 1000 people ranging from 0.3 per 1000 people in a typical remote district to 12.4 per 1000 population in a relatively urban district. Medical attendants, the highly unskilled cadre of health workers, constitute the largest proportion (40.2%) of the country’s total health workforce. Nurses form the largest proportion (27.8%) of the total skilled health workforce in Tanzania. The few health workers available are unequally distributed and many of them are concentrated in urban areas [11]. This mal-distribution is indicative of disproportionate access to quality health care services with rural populations being highly disadvantaged.

**Table 1:** percentage distribution of health workers across cadres and sectors in Tanzania (n=46,896)

**Cadre**	**Government**	**Private**	**Voluntary agency**	**Total**
Medical officer	0.8	0.3	0.2	1.3
Assistant medical officer	1.0	0.2	0.3	1.5
Clinical Officer	9.0	1.1	1.7	11.7
Nurse/Nurse midwife	18.3	2.1	7.4	27.8
Medical attendant	30.7	1.6	7.9	40.2
Other	10.6	1.5	5.3	17.5
**Total**	**70.3**	**6.7**	**22.9**	**100.0**

Source: [11].

### Task shifting pre-conditions

In the year 2010, the WHO had issued global recommendations on the steps to be considered by all countries which consider to scale up task shifting as one of the interventions to dealth with their health personnel crises. It was insisted by the WHO that these pre-conditions need to be adapted depending on countries’ specific circumstances [3]. These recommendations are listed below 

• Task shifting must be implemented in such a manner that it will improve the overall quality of care. It should not, and must not, be associated with second rate health care services

• Task shifting must be implemented with systems which contain checks and balances that are sufficient to protect both health workers and patients. This will involve both the presence of effective regulatory mechanisms, certification and remuneration of health workers who assumes new/delegated tasks

• Regulation and certification must neither decelerate the speed at which action is already taking place nor usher in restrictions that may have a constraining effect on other or on future public health service delivery effort

Albeit informally, task shifting in Tanzania is taking place in all health facility levels and it is practised both within cadres (medical officers, assistant medical offices and clinical officers) and between cadres (for example between clinical officers and nurses). Up to the time of writing this paper there was no rigorous scientific evidence permitting any conclusion regarding which tasks can easily be shifted from one cadre of health worker to another. Note that, there are many cadres of health workers in Tanzania and their tasks are described in detail in each cadre’s job description. It is thus difficult to present all tasks of all cadres in this single paper.

Many of the country’s health status indicators are not promising with HIV/AIDS and the fast re-merging non-communicable diseases posing as an additional burden to the already resource-constrained health care system. Task shifting practices in Tanzania are on-going and had been in place for many years [9]. To date, it is not surprising, for instance, to find an Assistant Medical Officer (AMO) working as a District Medical Officer (DMO), a post which is supposed to be held by a fully trained Medical Doctor (MD).

Geographically, Kongwa district (6°12′00″S 36°25′01″E6.200°S 36.417°E) where this study was conducted is one of the 5 districts constituting Dodoma Region. It is bordered to the North by Manyara Region, to the East by Morogoro Region, to the South by Mpwapwa District and to the West, by Dodoma Rural District. According to the 2002 Tanzania’s population and housing, the district has a population of about 249,760 inhabitants. We calculated the number of health workers per 1000 population based on the country’s HRH census of 2001/2002 and the 2002 population and housing census. We found that the district has 0.001 total health workers per 1000 population [12]. The district is among ten districts in the country with the lowest number of health workers per capita. In Tanzania, the legal framework and existing national health policies and regulations do not recognise task shifting (except for AMOs who are officially recognised to perform some of the MOs duties ) as an official strategy for addressing the health personnel crisis. However, there has been ongoing discussions and frequent stakeholder’s consultations focussing on how to adapt the WHO’s global recommendations which provide a recipe to be adhered before attempting to scale up task shifting.

## Methods

### Study design

In order to collect data needed to address our research questions and objectives, a qualitative study design was employed. We relied largely on information from Key Informant Interviews (KII) at the district level. Additional interviews were done with informants at the national level.

### Recruitment of key informants

Key informants at national and district levels were purposely selected on the basis of their strategic positions in policy and decision making processes related to HRH issues in Tanzania. Additionally, they were recruited based on their experience and their knowledge in specific policies and regulations regarding training, health worker carrier path (re)structuring and development. At the district level, five key informants were involved. They included such officials as the District Health Officer (DHO), the District Medical Officer (DMO), the District Nursing Officer (DNO), the District hospital Medical Officer in-charge and the District Human Resources Officer (DHRO). The initial plan was to include such informants as focal persons of the national disease programmes such as the District HIV/AIDS Coordinator (DAC), the district focal person for Malaria, Tuberculosis and Leprosy, and the in-charge of Reproductive and Child Health (DRCHC). However, after experiencing data saturation, the remaining interviews were discontinued. At the national level on the other hand, interviews were held with ten key informants, four of them were from the Ministry of Health and Social Welfare. The rest were from the following organisations with one informant each: Christian Social Service Commission, Ifakara Health Institute, World Health Organization, Medical Association of Tanzania, national AIDS Control Programme and the national Malaria Control Programme.

### Data collection: In-depth interviews

At the beginning of interviewing, a broad question capturing the study’s major issues was asked to each informant. This was followed by questions which sought clarifications on specific issues on task shifting. An interview guide with open-ended questions was used for this purpose. New issues relevant for the study were added to the interview guide and were further explored in the subsequent interviews. All the interviews were face to face and in Swahili which is the official language in Tanzania. However, in cases where the informant was not conversant with Swahili language, an English version of the interview guide was used. All the information collected through interviews was tape-recorded. The main topics in all interview sessions at the district level focused on district officials’ practical knowledge and experiences of the existing task shifting practices. Particularly, the interviews dwelt on district officials’ insights reflecting the actual implementation of task shifting and its implications for quality of services, work relationships between and within cadres of health workers, motivation, health worker productivity, coverage and its retention potentials. Additionally, interviews inquired on the prevalence of the practice and whether it happens as a deliberate policy strategy to counter the effect of shortage of health workers or it happens as an *adhoc* response to the crisis of health personnel due to limited options and resources. In addition to topics which guided interviews at the district level, national level interviews dwelt on policy issues related to long term strategies of dealing with the shortage of health workers. These policy issues included: implications of task shifting for the existing human resources policies related to remuneration, promotion, supervision, motivation, productivity assessment and training.

### Data analysis

All information collected through in-depth interviews was subjected to thematic content analysis. The process of analysing data was iterative and was concurrently done with data collection. The recorded data were transcribed and translated verbatim. The transcribed notes were combined with notes that were hand-written during data collection. Initial familiarization with the data was done at this stage. The research team used multiple-coding which involved the cross-checking of coding strategies and interpretation of data by different researchers. Four researchers were involved in this process. Multiple coding was done to create coding categories which were capable of reflecting the content of the data and concepts used by the informants rather than the questions or concepts predetermined in the interview guide. The coding categories extracted from the transcripts were used to systematically analyse commonalities and apparent contradictions reflected in the data by focusing on issues which were repeatedly mentioned or strongly emphasized by the informants. However, we did not observe any obvious and significant contradictions between views expressed by the national and district level informants.

### Ethical issues

This was a non-intrusive study. However, ethics clearance was requested from and was issued by the Medical Research Coordinating Committee (MRCC) of the National Institute for Medical Research (NIMR) prior to actual data collection. Informed consent was sought from the study informants after having explained the aims of the study and assuring them that their participation was voluntary and no personal identifiers will be made available to anyone outside the research team.

## Results

In this section, we start by providing a description of informants’ awareness, acceptability and the extent to which task shifting is implemented as a strategy to deal with acute shortages of health personnel. We then present informants’ perceptions on the potential benefits, opportunities and challenges associated with implementation of task shifting.

### Awareness, acceptability and the extent of task shifting as a coping strategy to counter the shortage of health workers

Both at national and district levels, informants registered broad consensus regarding their awareness of the on-going task shifting practices in the country. In relation to this common understanding among informants, informants at the national level warned that task shifting is not mandated by laws governing medical practice in Tanzania and hence not fully realized. They pointed out that informal task shifting is more prevalent in rural areas than in urban areas where for example Assistant Medical Officers (AMOs) perform many clinical tasks that would otherwise be performed by Medical Officers.

“Task shifting in Tanzania is prevalent in health care delivery. In most cases medical attendants are doing tasks of nursing officers much as clinical officers perform medical officers’ tasks (National level informant).

*“In fact, Tanzania is one of the pioneers of task shifting whereby the cadre called Assistant Medical Officer (AMO) had been officially recognized by the ministry of health for quite long time now. In this country, about 80% of all caesarean sections are being performed by AMOs, the tasks which should otherwise be performed by Medical Doctors or specialists* (National level informant).

*“We have a 40% deficit of clinical officers in this district who are needed to provide services to our dispensaries and health centres. Because of this shortage much of the clinical work which is supposed to be the responsibility of clinical officers, is manned by medical attendants.”* (District level informant)

### Feasibility of task shifting and the associated requirements

Based on their experiences, respondents were asked to air their opinions on the feasibility of task shifting. Majority of informants both at the national and district levels conceded that task shifting is feasible but any decision to scale it up has to seriously consider issues of quality of services and other associated challenges of implementing this initiative. They pointed out that with simple and standardised procedures for managing simple clinical procedures, and strong supervision and mentoring, task shifting can produce some good results. However, designing long term and sustainable intervention to address the health personnel crisis, was widely emphasised.

*“If we are to take a pragmatic approach, then our focus should be on producing more nurses and clinical officers and the associated mid-level cadres because these are the types of cadres who are more likely or more willing to work and accept to stay in underserved areas and the cost of producing these cadres is relatively cheaper than that of producing higher level cadres such as medical doctors and specialist” *(National level informant).

### ‘Vertical’ versus ‘horizontal’ task shifting

Regarding types of task shifting, Manongi *et al.* had coined ‘vertical’ and ‘horizontal’ task shifting as most common concepts associated with task shifting in Tanzania [13]. Analysis of our data presented in this paper provides a confirmation to these concepts, though from a different study site. The above authors had further sub-divided ‘vertical’ task shifting into ‘upward’ and ‘downward acting’ to reflect how it is actually practised. Our ‘Upward’ acting is a scenario whereby for whatever reasons, lowly trained health personnel (or untrained ones) take the duties of properly trained health personnel or higher cadre health workers. Downward acting on the other hand involves a scenario whereby, for whatever reasons, a highly trained health worker takes the duties of a lower level cadre or unskilled health worker. ‘Horizontal’ task shifting is also practised, although is more widespread than ‘vertical’ task shifting. It involves a situation whereby in the context of shortage of appropriate personnel, physician’s clinical tasks are delegated to non-physicians, for example from clinical officers to nurses or laboratory personnel. This was also reported to be prevalent in our study site. The quotes below confirm the earlier finding and attest that upward and downward acting were also prevalent in our study setting.

“We have a 40% shortage of clinical officers in our district. According to the government regulations, every government owned dispensary is supposed to be led by a clinical officer who is the in-charge of clinical and administrative tasks at this level. But due to this shortage, many dispensaries are manned by medical attendants, with very limited medical skills (District level informant).

*“District Medical Officers (DMOs) are by law required to have qualified as Medical Doctors. However, there are quite a good number of district hospitals in Tanzania whereby the DMO is only qualified as an Assistant Medical Officer (AMOs)” *(National level informant)

### Regulatory policies in relation to implementation of task shifting

By implications, the responses from almost all our informants at national and district levels indicated that there are no policies or regulations to guide implementation, monitoring and evaluation of task shifting practices in Tanzania. This is regardless of the fact that all cadres have clear job descriptions. However, due to acute shortages of qualified health workers’ job descriptions are not adhered to.

*“I am not sure about the existence of regulatory policies, but what I know is that AMOs can perform gynaecological operations although they are not supposed to do so but due to shortage of workers with such specialised qualifications, they always perform such procedures. For instance in Muhimbili National Hospital, medical attendants are not allowed to offer injections. We know they are not allowed, but what else can you do if you were in such a situation? let people die?”* (National levelinformant).

### Task shifting and quality of care

Regarding the presence of quality assurance policies and guidelines, respondents said that provision of all health services in the country is regulated by the National Health policy and the associated guidelines. This is also supported by the activities of health professional councils and associations which set and oversee ethical standards of care are abided by all health workers. They further indicated that it is currently not explicit whether quality assurance frameworks are supposed to be embedded in the implementation of task shifting because the practice is implemented informally. However, the practice has been in place since 1960s probably because it is an ‘inevitable’ coping strategy of dealing with the HRH crisis in the country. Related to the ‘inevitability’ of task shifting in the Tanzanian context, one respondent put it this way:

*“ In circumstances where a seriously sick patient come to a dispensary, the only health worker who is present at that facility at that time cannot just let the patient die. He or she must do something even if there is no doctor to attend him. This is really challenging, especially in relation to the quality of services expected by clients and that which is predetermined by national policies and guidelines”* (National level informant).

While almost all informants at the district and national level were seriously worried about the potential negative effect on quality of services as a result of implementing task shifting, few others were concerned about the limits for which workers are supposed operate. That is which tasks can be delegated and be implemented effectively.

*“The quality of service will be impaired if the workers are not clear on what needs to be done and to what level and by whom. *(National level informant)

Further to the above contentions, bold recommendations were consistently provided by our informants pointing to policy makers and planners to make sufficient preparations before considering to up-scale task shifting.

*“ No I do not think that we should rush to scale up task shifting before working on establishing a strong foundation for its implementation. I personally think that this is a short term remedy. Long term and sustainable strategies should consider expanding training capacity of our training institutions and ensure that only the skilled staffs come into service. Lower level cadres will also need to gradually upgrade their professional skills through the implementation of effective carrier advancement programmes and policies which support continuing education and on-the-job training” *(National level informant).

‘If we are not careful, task shifting will relax our efforts to design effective interventions that are needed to offer long-lasting solution to the current health personnel crisis (District level informant).

### Task shifting and the potential for ‘professional war’

Many informants at both the national and district levels had the perception that implementation of task shifting by legally incorporating the tasks of physician-clinicians to non-physician-clinicians or other lower cadres may not only be counterproductive to the quality of health services, but may also undermine the ‘professional distinction’ of those who have spent many years to earn such professions. In this regard, one informant commented:

“I will not support to legalise it as it will cause professional commotion and conflicts between workers of different cadres and different levels of training. This may seriously affect the quality of services delivered to patients” (National level informant)

### Opportunities and perceived benefits of implementing task shifting

Almost all informants at the national level and majority at the district level conceded that the historical context of Tanzania provides an opportunity that task shifting is feasible. In this regard, they directly made reference to the experience of using AMOs to perform tasks which would otherwise be performed by MOs. The following subsections describe other perceived opportunities and benefits of implementing task shifting in the context of health personnel crisis in Tanzania.

#### A sense of pride as one assumes superior tasks

Informants at the district level had generally conceived that under supportive supervision and effective mentoring task shifting can be a motivational factor especially when lower cadre health workers are trusted and assigned responsibilities which would otherwise be performed by higher cadre personnel. Additional incentives were perceived as capable of reinforcing this ‘potential’ motivation and ultimately increase health workers productivity. In the same way, additional supervisory roles could work two ways, both as a motivational factor for those entrusted with new supervisory roles but also a disincentive especially in a situation of serious shortages of workers and limited incentives as the case was in this study. One informant at the national level insisted that:

“It (task shifting) motivates and create challenging environment especially if high level tasks are delegated to lower level cadres. It creates excitement in the case of the job well done among those to whom tasks are delegated.” (National level informant).

“It may de-motivate the cadre or health worker, from whom the tasks are shifted from, making them feeling useless or disregarded. Additionally, health workers may feel undermined upon receiving advice from a lower cadres, for instance a nurse advising a clinical officer on how to manage a certain clinical procedure-that will definitely annoy the latter” (National level informant).

#### Enhancing rural retention of health workers

Increased retention of health workers especially in remote areas was perceived by the majority of as one of the potential benefits to be accrued out of implementing task shifting strategy. Informants had a consistent perception that it is easier to maintain a lower cadre health worker who is already working in the remote health facility than deploying qualified personnel from somewhere else, particularly in urban areas. This was perceived to be true both from the point of view related to recruitment costs and the fact that the existing health workers (lower cadre) are already used and immersed in the life conditions of remote areas.

*“Skills of lower cadre health workers and especially community health workers are hardly portable both nationally and internationally. Lower cadre health workers can also be easily and cheaply recruited from within areas where they live and where they are supposed to be working. It is thus easy to retain these workers as they are already used to the living conditions of their localities” *(District level informant).

#### Coverage and access to health services

Increased coverage and access to health services was perceived by majority of informants both at the national and district level as one of the potential benefits of implementing task shifting in a resource constrained settings like Tanzania.

“Consider a situation of a typical remote dispensary where you only have one medical attendant doing everything- medical examination, drug prescription to managing injections and drug dispensing! His absence implies that even small procedures such as managing simple interventions would not have happened and many people would have been denied access to services, notwithstanding considerations of quality. I must assure you that, in most of remote villages, services such as family planning are managed by lower cadre health workers such as medical attendants and community health workers” (District level informant).

### Challenges related to implementing task shifting

#### Training and supervision requirement

Almost all Informants at district and national levels indicated that in most cases, tasks are delegated without any special or additional training to those who are supposed to implement them. However, there are sporadic trainings given to some health workers especially by disease-specific programmes such as those addressing HIV/AIDS, Malaria and Tuberculosis. These types of trainings only happen when these programmes have some skills they want to impart to health workers to perform specific tasks, for example general HIV/AIDS counselling training given to nurses. In most cases, delegation of tasks is done based on health workers’ experience in the absence of clear policy guidelines. In terms of supervision, it was learnt that implementing task shifting may not only be costly but also time consuming.

“Since implementing task shifting requires special supervision in order to ensure that quality of service is not compromised, it will be costly and time consuming for the few available skilled staff as most of their time will be spent to supervise lower cadres or unskilled workers ” (National level informant)

#### Aligning new tasks and the job description: implications for promotion and career development

Health personnel policy issues were also raised especially by majority of informants at the national level. These issues were related to how new tasks from higher cadres or other cadres or skilled professionals to lower/other/unskilled cadres could be aligned with their job descriptions and what implications this has for their promotion and career development. A quite valid concern was raised by informants both at the national and district level regarding implementation of task shifting in the current human recourses policies which guides employees promotion and career development. Related to this, the following quote illustrates:

“If task-shifting exercise lacks a framework of job description and performance measures as the situation currently is, then the development and promotion of a health workers will be affected as there will be no fair and clear criteria to be followed in the process of deciding who deserves promotion and who does not ” (National level)

## Discussion

This study is one of few attempts in Tanzania describing the current situation of task shifting in the health sector. It has especially focused on task shifting’s inevitability due to serious shortages of health workers in remote and mostly underserved areas. It has highlighted some of the potential opportunities offered by task shifting in terms of improving coverage of health services and rural retention of health workers. It also brought forward the discussion on possible challenges that may come with implementation of task shifting in relation to quality of health care, management of human resources policies as they relate to training and development, the need for additional incentives, motivation and staff promotion. Hereunder, are sub-sections discussing the main findings and methodological limitations.

### Task shifting is inevitable, nevertheless a short term solution

Almost all of our informants have conceded that task shifting should be taken as a short term solution while the government is working on more effective and sustainable ways of arresting the health personnel crisis in the country. From our study informants, it has been evident that the practice of task shifting is prevailing in almost all health facilities ranging from the district hospital to the dispensaries. This finding is not surprising, given its inevitability especially in situations of enormous shortages of health workers both in terms of numbers and the required skills [3]. In relation to the implementation of task shifting, it has been had argued that: *“*informal task shifting which happens as an *ad hoc* response to need rather than as an explicit policy strategy, may result in the proliferation of new cadres with vague or overlapping responsibilities, which are then questioned by the existing staff, policy makers and the patients themselves” [5]. Note that, our findings indicate that the practice of task shifting has been perceived by our informants as acceptable. However, this perception might be due to unavailability of the required health workers making the practice to be one of the ‘inevitable’ coping strategies to address the shortage of health personnel, notwithstanding its *ad hoc* nature of implementation. We are tempted to believe that the inevitability of task shifting could be one crucial reason for the increased momentum and efforts by both national and international stakeholders to chart out the optimal ways on how to scale it up. As these efforts and discussions continue rapidly; and the need to scale up task shifting becomes apparent, a number of lessons can be drawn from the field of HIV/AIDS service delivery [2,5]. Some of these lessons include the need to clearly define the limits of task shifting, ensure adequate training and supervision, provide adequate remuneration and recognition, develop simplified tools and guidelines, develop and implement simplified recording and reporting systems, develop simplified monitoring and evaluation procedures, ensure engagement with regulatory bodies while at the same time, mobilise community members, other stakeholders and resources in order to make task shifting sustainable [3,5,7]

### ‘Vertical’ vs ‘horizontal’ task shifting

This study has provided confirmation of two types of task shifting namely; ‘horizontal’ and ‘vertical’ task shifting as coined and interpreted by different authors [3,5,13]. Comparably, vertical task shifting has been perceived by majority of our informants as easier to be implemented in the Tanzanian context than horizontal task shifting. The main reason for this perception is that it is easier to train a cadre of health workers who belong to the same ‘family’ of professional practice, for example physician-clinicians. Meaning that, it is easier to delegate task from higher level physician-clinicians to lower level physician-clinician than to delegate the same tasks to non-physician clinicians. The logic of this perception lies in the level of effort and resources needed to train and supervise those to whom new tasks and responsibilities have been delegated. The fact that delegating tasks and responsibilities of specialists to non-specialists requires additional training and supervision has been acknowledged by previous reports [3,5,13]. However, there is virually no published scientific evidence on the costs and cost- effectiveness of task shifting implementation in resource limited settings such as Tanzania. Future studies should thus focus on addressing this evidence gap.

### Task shifting and the potential for increasing access and coverage to health services in underserved remote areas

This study has demonstrated that task shifting has a potential for increasing coverage and access to health services especially in rural and remote areas which faces a disproportionally huge shortages of health workers. Similar findings have been documented elsewhere [5,14]. In the context of offering caesarean section services, Friedman had argued that task shifting to less trained health workers can contribute to substantial increase in access to services and reduction of maternal mortality [14]. In Tanzania and especially in the delivery of simple health care interventions, many health workers with lower skills have been performing tasks way out of their professional training and job descriptions [15].Community drug distributors have been recommended and successfully been used in different health care settings to manage simple health service interventions [3,5,14]. Similarly, the use non-physician clinicians in Malawi had significantly contributed to the successful roll-out of the Malawian national ART plan [16,17]. From the above discussions, it is noteworthy that task shifting seems to be feasible and effective only in the delivery of ‘simple’ health interventions which logically require less additional training and efforts needed for additional supervision.

### Task shifting and the potential for improving retention of health workers in remote areas

Retention of health workers in remote and underserved areas is a critical policy issue for Tanzania as it is for many low and middle income countries. This study indicated that task shifting has the potential for improving the retention of health workers in remote areas where the needs for health care services are higher than the available health personnel. With some optimism, respondents emphasised that in order to exploit task shifting as a positive incentive for rural retention of health workers, its implementation should be accompanied or rather reinforced by additional incentives and effective supportive supervision. If the above mentioned prerequisites can be realised (given the fact that many countries are resource-constrained) it is then likely that remote-specific cadres of health workers can be established and retained in remote areas. Note that, the qualifications of the said remote-specific cadres are not easily portable and thus it is somewhat difficult for them to opt out for rural–urban or international migration. A report by Zachariah *et al.* had highlighted similar observations by arguing further that, task shifting can be expected to support the retention of existing health workers by reducing burnout and increasing morale through more efficient team management of patient case-loads [5]. A follow up study of a task shifting programme conducted in Mozambique by Pereira *et al.* had found that after 7 years of engaging non-physician clinicians in the provision of obstetric care, about 90% of non-physicians were still working in the district while almost all of the medical doctors available had left [18]. Our findings and observations from the literature might have two policy implications. Firstly, in order to have an effective task shifting programme, policy makers should consider paying health workers decent salaries which constitute living wages and which are commensurate with their responsibilities. This payment which should be linked to expanded responsibilities must also be associated with healthy working environments which may include additional incentives such as provision of a reasonably decent housing, provision of opportunities for educational and professional development and sustained supportive supervision programmes. Secondly and most importantly, is the consideration by all stakeholders that planning for effective task shifting strategy which is expected to lead to improved retention of health workers should be *comprehensive* and *multi-sectoral*. Task shifting as an innovation to improve retention of health workers is recommended to be comprehensive and multi-sectoral because oftentimes, ineffective retention strategies characterising many health systems in low and middle income countries are not due to malfunctioning of the ministries or departments of health alone, many stakeholders out of the health sector have important stakes too in the affairs of health sector [3,19,20].

### Task shifting and quality of health services

Findings from this study demonstrate that relinquishing tasks which involve complex procedures to completely unskilled or lower cadre personnel may be counterproductive not only to the patients’ general health conditions but also may threaten their lives. Our informants recommended that, in order to ensure that implementation of task shifting does not compromise quality of health care services which is already very poor; there is a need to have clear and acceptable job descriptions which will guide on how to define task limits. Clear job descriptions alone are not enough to effectively delegate tasks from more skilled to less skilled health workers. They should be accompanied by adequate financial and non-financial incentives. Note that, financial and non-financial incentives can be used as a means for retaining and enhancing productivity of health workers with new or additional responsibilities [3,5,21]. However, the need for additional incentives as a prerequisite to effectively implement task shifting is quite an obvious challenge especially in a country experiencing serious crisis of shortage of economic and financial resources such as Tanzania.

Other findings from the literature had originally presented a more or less similar picture as presented by our study regarding the implications of implementing task shifting for quality of health services. Chu *et al.* have contended that shifting clinical responsibilities from higher to lower cadres raises ethical concerns of lowering standards of care [7].In the current study, this ethical concern featured very explicitly as most of our informants consistently associated the practice of task shifting with declining quality of services might. It has been recommended that, in order to ensure that task shifting deliver quality health care services, its implementation must be aligned with the broader health systems strengthening [8,21,22]. This is due to the fact that task shifting practice is indeed not a solution to a weak health system. Other long term and sustainable strategies should be implemented in line with task shifting [8,21,22]. That is, health systems must assure a number of functions such as clinical care, uninterrupted supply, training and supervision, improved remuneration to health workers are effectively implemented before considering to scale up task shifting [8,21,22].

### Task shifting implementation amidst absence of monitoring and evaluation tools

Absence of regulatory frameworks to suit the demands of monitoring quality aspects associated with the implementation of task shifting practices was also mentioned by our informants as one of the possible reasons which may compromise quality of health care. This finding confirms what has already being reported [3,5]. In Tanzania for example, there are known professional and ethical regulations which guide the practice of such cadres as MOs and nurses. Other cadres’ professional and ethical conducts are regulated or managed by the existing rules and regulations of the establishment and those issued by the Ministry of Health and Social Welfare. This implies that, regulations (from professional organisations, mainly covering nurses and doctors) may not be meaningful or satisfactory for monitoring quality of a health services provided by other health workers who are outside the two cadres (doctors and nurses). This further means that, if there will be a reason to scale up task shifting given accumulated evidence on its feasibility and cost-effectiveness, then the existing professional and ethical regulations need to be intensively reviewed to give room for the new demands associated with the implementation of task shifting.

### Task shifting: its implications for motivation (or de-motivation) and associated productivity

It has been shown that when properly managed, task shifting, and could act as an incentive for motivating health workers to exert more effort in delivering services to their clients. Results have indicated that if superior tasks are delegated to a lower skilled health worker, there is potential for increased motivation as those to whom tasks are delegated will feel a sense of pride and recognition. The literature is however a little bit vigilant on suggesting a direct and clear link between task delegation and increased motivation. In the context of task shifting, motivation is expected to happen only when the increased tasks are accompanied with additional incentives, supportive supervision and good working environment [3,5,6,21,23]. One randomised study has indicated that supportive supervision (not just that pertaining to task shifting) can increase health worker productivity at least in the short term. It has also been argued that, if properly done, supervision can act as a mechanism for providing professional development (for both supervisors and supervisees), improving health workers’ job satisfaction and ultimately increasing motivation and thus, productivity [24,25]. On the contrary, task delegation may at times be a de-motivating factor. Our informants have indicated that additional supervisory roles to the fewer skilled and experienced health workers available may act as a disincentive if there are no supportive working environments. The literature suggest that too often, supervisors lack useful skills, working tools and transportation; and are usually overburdened by a number of administrative duties [26,27]. Thus, it is unnatural to expect that health worker motivation and productivity will increase only because tasks are decentralised. Good working environment is an equally important input for increasing performance, with or without task shifting.

### Strengths and limitation of the study

This study was purely qualitative and exploratory in nature and thus unable to provide conclusive policy recommendations. However, the study has provided a strong research foundation whereby appropriate research questions can be formulated and followed up by using well designed experimental studies to verify some of the important findings reported in this and other studies. For example, in the context of differing levels of financial resources between HIV/AIDS care and overall health care delivery system, quality aspects can be assessed using the same cadres of health workers (for example nurses performing tasks which would otherwise be performed by clinicians) by comparing them when they operate in the HIV/AIDS specific care and when they are offering other health services. Findings from such studies might increase analysts’ confidence in their conclusions as regards to whether task shifting compromises or improves quality of health care services. Thus, this somewhat lustrous task-shifting’s picture seen in the HIV/AIDS care should be re-examined so as to see whether the same picture can be painted in the delivery of other non-HIV/AIDS services.

### Conclusions

This descriptive qualitative study adds little to the existing knowledge base on task shifting practices in a typical underserved setting of a developing country. However, it strongly supports the assertion by many stakeholders that, task shifting should only be taken as a short term solution. Otherwise, if it is embraced as a magic bullet, efforts needed to offer long-term solutions will be relaxed. Task shifting is already in practice and has been taken by health managers as a rapid response to acute shortages of human resources in many health care settings. Its implementation occurs only as an *ad hoc* coping mechanism to the existing human resources crisis rather than a deliberate policy response to the crisis. In order to rectify this situation, this research suggests the following policy actions:

• As a first step, future research should focus on gathering and synthesising enough evidence in order to ascertain the optimal ways of task shifting implementation and provide a framework for monitoring and evaluation its effectiveness, its impact on quality and costs. In addition, future research should focus on quantifying the extent to which task shifting is practiced in health care systems by analysing the gaps between what is prescribed in health workers’ job descriptions and what they actually do.

• The second step should require health managers and policy analysts to not only determine whether task shifting is cost effective, but also define the limits of task shifting and determine where it can have the strongest and most sustainable impact in the delivery of quality health services.

• Finally, health managers should mobilise important stakeholders and resources and engage them in broader, evidence-based- discussions on pertinent issues regarding the best ways to employ task shifting (albeit as a short term solution to the existing health personnel crisis) while at the same time ponder on the longer term and sustainable interventions needed to alleviate the health workers’ crisis.

### Endnote

^a^The identified possible programmes included national HIV/AIDS, Malaria, Tuberculosis

## Competing interest

The authors declare that they have no competing interest.

## Authors’ contribution

Michael Aloyce Munga conceived the idea, prepared the proposal and submitted it for funding, participated in data collection and analysis and had the sole responsibility of preparing the first drafts of this paper. Other authors have equally participated in data collection, analysis and critically commented on the manuscript. All authors have read and agreed the manuscript to be submitted as it is.

## Pre-publication history

The pre-publication history for this paper can be accessed here:

http://www.biomedcentral.com/1472-698X/12/27/prepub
